# N-acetyl-cysteine exhibits potent anti-mycobacterial activity in addition to its known anti-oxidative functions

**DOI:** 10.1186/s12866-016-0872-7

**Published:** 2016-10-28

**Authors:** Eduardo P. Amaral, Elisabete L. Conceição, Diego L. Costa, Michael S. Rocha, Jamocyr M. Marinho, Marcelo Cordeiro-Santos, Maria Regina D’Império-Lima, Theolis Barbosa, Alan Sher, Bruno B. Andrade

**Affiliations:** 1 Immunobiology Section, Laboratory of Parasitic Diseases, National Institute of Allergy and Infectious Diseases, National Institutes of Health, Bethesda, MD 20892 USA; 2 Department of Immunology, Laboratory of Immunology of Infectious Diseases, Institute of Biomedical Science, University of São Paulo, São Paulo, 05508-900 Brazil; 3 Laboratório Integrado de Microbiologia e Imunorregulação (LIMI), Instituto Gonçalo Moniz, Fundação Oswaldo Cruz (FIOCRUZ), Salvador, 40296-710 Bahia Brazil; 4 Instituto de Ciências da Saúde (ICS), Universidade Federal da Bahia, Salvador, 40110-100 Brazil; 5 Departament of Internal Medicine, School of Medicine and Public Health, Salvador, 41150-100 Brazil; 6 Programa de Controle da Tuberculose, Hospital Especializado Octávio Mangabeira, Salvador, 40320-350 Brazil; 7 Departamento de Ensino e Pós-Graduação, Fundação de Medicina Tropical Dr. Heitor Vieira Dourado, Manaus, Brazil; 8 Programa de Pós-Graduação em Medicina Tropical, Universidade do Estado do Amazonas, Manaus, Brazil; 9 Multinational Organization Network Sponsoring Translational and Epidemiological Research (MONSTER) Initiative, Fundação José Silveira, Salvador, 45204-040 Brazil; 10 Curso de Medicina, Faculdade de Tecnologia e Ciências, Salvador, 41741-590 Brazil

**Keywords:** Tuberculosis, N-acetyl cysteine, Antimicrobial activity, Therapy

## Abstract

**Background:**

*Mycobacterium tuberculosis* infection is thought to induce oxidative stress. N-acetyl-cysteine (NAC) is widely used in patients with chronic pulmonary diseases including tuberculosis due to its mucolytic and anti-oxidant activities. Here, we tested whether NAC exerts a direct antibiotic activity against mycobacteria.

**Methods:**

Oxidative stress status in plasma was compared between pulmonary TB (PTB) patients and those with latent *M. tuberculosis* infection (LTBI) or healthy uninfected individuals. Lipid peroxidation, DNA oxidation and cell death, as well as accumulation of reactive oxygen species (ROS) were measured in cultures of primary human monocyte-derived macrophages infected with *M. tuberculosis* and treated or not with NAC. *M. tuberculosis*, *M. avium* and *M. bovis* BCG cultures were also exposed to different doses of NAC with or without medium pH adjustment to control for acidity. The anti-mycobacterial effect of NAC was assessed in *M. tuberculosis* infected human THP-1 cells and bone marrow-derived macrophages from mice lacking a fully functional NADPH oxidase system. The capacity of NAC to control *M. tuberculosis* infection was further tested in vivo in a mouse (C57BL/6) model.

**Results:**

PTB patients exhibited elevated levels of oxidation products and a reduction of anti-oxidants compared with LTBI cases or uninfected controls. NAC treatment in *M. tuberculosis*-infected human macrophages resulted in a decrease of oxidative stress and cell death evoked by mycobacteria. Importantly, we observed a dose-dependent reduction in metabolic activity and in vitro growth of NAC treated *M. tuberculosis*, *M. avium* and *M. bovis* BCG. Furthermore, anti-mycobacterial activity in infected macrophages was shown to be independent of the effects of NAC on the host NADPH oxidase system in vitro. Short-term NAC treatment of *M. tuberculosis* infected mice in vivo resulted in a significant reduction of mycobacterial loads in the lungs.

**Conclusions:**

NAC exhibits potent anti-mycobacterial effects and may limit *M. tuberculosis* infection and disease both through suppression of the host oxidative response and through direct antimicrobial activity.

**Electronic supplementary material:**

The online version of this article (doi:10.1186/s12866-016-0872-7) contains supplementary material, which is available to authorized users.

## Background

N-acetyl-cysteine (NAC) is included in the World Health Organization’s list of essential medicines; a list that details the most relevant medications needed for a basic health system [1]. Acetyl-cysteine is a derivative of cysteine in which an acetyl group is attached to nitrogen. Due to its disulfide reducing activity, NAC is used as a mucolytic agent to promote expectoration [2]. NAC is commonly prescribed as an adjunct therapy in patients with a wide range of respiratory diseases characterized by formation of thick mucus, such as cystic fibrosis [2–4]. At high doses, NAC results in significantly improved small airway function and decreased exacerbation frequency in patients with stable chronic obstructive pulmonary disease (COPD) [3, 4]. NAC’s mucolytic activity is also the basis of its use in liquefying sputum samples for the microscopic detection of acid-fast bacilli (AFB) in suspected pulmonary tuberculosis (TB) patients [5]. Furthermore, in both experimental animal models and clinical studies, NAC displays a protective effect on acute liver injury induced by anti-TB drugs in acetaminophen-dependent or independent conditions [6–11]. In patients with type 2 diabetes, NAC holds promise in primary prevention of cardiovascular complications and systemic inflammation [12–14].

In addition to the above clinical applications, NAC has been employed as a potent anti-oxidant in several experimental models of infection and cancer in vitro and in vivo [15–20]. In these settings, NAC serves as a pro-drug to L-cysteine, which is a precursor to the biologic antioxidant glutathione. This anti-oxidant property of NAC is associated with strong anti-inflammatory effects, which have been suggested to inhibit the activation of nuclear factor-κB (NF-κB) with subsequent inhibition of cytokine synthesis [2, 21, 22]. In a mammalian model of *Mycobacterium tuberculosis* infection, NAC has been shown to diminish TB-driven lung pathology and inflammatory status, as well as to reduce mycobacterial infection loads in the lung [23]. These effects were attributed to the drug’s anti-oxidant properties and immune regulatory activities. Whether NAC limits *M. tuberculosis* infection in this situation through a direct microbicidal effect on *M. tuberculosis* was not addressed. Indeed, NAC has been shown to exhibit anti-microbial activity against a number of bacterial pathogens including *Pseudomonas aeruginosa, Staphylococcus aureus, Helicobacter pylori, Klebsiella pneumoniae* and *Enterobacter cloacae* [17, 24–26].

In this study, we demonstrate that NAC directly impairs the growth of several species of mycobacteria in vitro independent of its inhibitory effects on the host NADPH oxidase system. This anti-mycobacterial effect was also observed in an experimental model in vivo. Thus, NAC may limit *M. tuberculosis* infection and disease both through suppression of the host oxidative response and through direct antimicrobial activity. This dual host and pathogen directed function makes the drug an interesting candidate for use as adjunct therapy for tuberculosis.

## Methods

### Clinical study

Cryopreserved heparinized plasma samples were collected from 30 subjects with active pulmonary TB (20 males; median age 32 years, interquartile range [IQR]: 18–47), 20 individuals with LTBI (10 males; median age 31 years, IQR: 22–46) and 20 healthy controls (8 males; median age 20 years, IQR: 19–38). The study groups were similar with regard to age (*p = 0.248*) and gender (*p = 0.162*). Subjects were recruited between May and November 2012 at the Hospital Especializado Octávio Mangabeira, Salvador, Brazil, as part of a biomarker study [27]. Tuberculosis diagnosis included positive AFB in sputum smears and positive *M. tuberculosis* sputum cultures. Three sputum samples per subject were examined by fluorescence microscopy, processed by the modified Petroff’s method and cultured on Lowenstein-Jensen medium. LTBI diagnosis was performed in contacts of active TB cases who agreed to participate in the study. Diagnosis was based on tuberculin skin test (TST) positivity (≥10 mm in diameter), absence of chest radiography abnormalities or pulmonary symptoms and negative sputum cultures. Healthy control subjects (health care professionals and medical students from the Hospital Especializado Octávio Mangabeira who agreed to participate) were asymptomatic with normal chest radiograph and negative sputum cultures and TST induration (<5 mm in diameter). At the time of enrollment, all individuals were HIV negative (all patients were actively screened), BCG-vaccinated and had no record of prior TB disease or of anti-TB treatment.

### Mice

C57BL/6 mice, male, 6–8 weeks old (*n* = 20), were purchased from Taconic Farms (Hudson, NY). The *gp91Phox* ^*−/−*^ mice, male, 6–8 weeks old (*n* = 10), genetically backcrossed in the C57BL/6 background were a kind gift from Dr. Sharon Jackson (NIMHD, NIH). These mice do not exhibit a fully functional NADPH oxidase system [28, 29]. All mice were maintained in micro-isolator cages under controlled temperature and humidity. They were fed ad libitum at NIAID animal facilities.

### Bacteria

The H37Rv *M. tuberculosis* (ATCC)*,* Beijing 1471 hypervirulent *M. tuberculosis* [30], *Mycobacterium avium* SmT 2151 and *Mycobacterium bovis BCG,* strains were used. Mycobacteria from a single colony-forming unit (CFU) were suspended in Middlebrook 7H9 medium (Difco, BD Biosciences, USA) that was supplemented with 10 % albumin-dextrose-catalase (ADC; Difco) and 0.05 % Tween 80 (Sigma-Aldrich, USA), cultivated and then frozen at −80 °C in aliquots of 10^8^ bacilli/mL. Prior to performing the experiments, the aliquots were thawed and diluted in complete 7H9 medium. To avoid bacterial clumps, the samples were sonicated for 30 s and homogenized. The bacilli were quantified by spectrophotometry at 600 nm. In some experiments, the pH in Middlebrook 7H9 medium was adjusted in acidic conditions (pH 5.8) and neutral conditions (pH 6.8–7.4) as indicated in the figures.

### Cell cultures

CD14^+^ column-purified human elutriated monocytes were obtained from peripheral blood of healthy donors at the NIH blood bank. Macrophages were generated by culturing monocytes in the presence of RPMI media containing 10 % human AB serum and M-CSF 50 ηg/mL (Prepotech, Rocky Hill, NJ) for 7 days, with fresh media with growth factor being added every 48 h as previously described [31]. For infection assays, cells were plated at the concentration of 10^6^ cells/well in 24-well plates in phenol and serum free media (Opti-MEM; Life Technologies, Carlsbad, CA).

The human monocyte-like cell line (ATCC) THP-1 was differentiated into mature macrophages by treatment with phorbol ester (PMA; Sigma Aldrich, USA). Briefly, THP-1 cells were cultured in RPMI medium (Gibco; 1 mM sodium pyruvate, 2 mM glutamine) supplemented with 10 % fetal calf serum at 37 °C in 5 % CO_2_. For the experiments, THP-1 cells were seeded on 96-well plate at 10^5^ cells/well in the presence of PMA (40 ηmol/L) for 24 h to induce macrophage differentiation [32, 33]. Cells were washed and incubated for additional 24 h in fresh medium without PMA until their use in experiments. After infection with mycobacteria, the macrophage cultures were washed to remove extracellular bacteria and then cells were cultured in phenol and serum free media (Opti-MEM).

To obtain mouse bone marrow derived macrophages (BMDM), bone marrow cells were cultivated in 30 % L929 cell-conditioned medium for 7 days as previously described [34]. An additional 10 ml of L929 cell-conditioned medium were added after 4 days of incubation. BMDMs were detached with cold PBS and seeded in 96-well plates at 10^5^ cells/well, containing serum and phenol free media at 37 °C in 5 % CO_2_.

In experiments testing the effects of NAC on mycobacterial infection in vitro, cells (BMDM or THP-1 macrophages) were infected with H37Rv, *M. avium* or *M. bovis* BCG at MOI of 10 for 3 h, washed and then cultivated for 5 days. Bacterial uptake was evaluated at different time points by measuring CFU in the infected BMDM cultures following treatment with 0.05 % saponin (Sigma-Aldrich, USA) for 10 min [30].

### Mycobacterial quantification

Bacterial numbers were determined by serial dilution of bacterial cultures and cell lysates in Middlebrook 7H11 medium (Difco) supplemented with 10 % oleic acid/albumin/dextrose/catalase (OADC; Difco). CFU numbers were determined after 3 weeks of incubation at 37 °C in 5 % CO_2_.

### Determination of mycobacterial metabolic activity

Metabolic activity of bacilli was evaluated using a tetrazole salt assay in a 96-well plate as described previously [35]. Briefly, bacteria were pipetted in a 96-well plate at 10^6^ CFU/well (50 μL), and NAC (50 μL) diluted in Middlebrook 7H9 added at the indicated concentrations. Plates were then incubated at 37 °C in 5 % CO_2_ for 5 days of indicated time points. After this period, tetrazolium salt (3-[4,5-dimethylthiazol-2-yl]-2,5-diphenyltetrazole) at 5 mg/mL (final concentration) was added to the cultures for 3 h. Next, 100 μL of lysis buffer (20 % w/v SDS/50 % DMF – dimethylformamide in distilled water) was added and the cultures were incubated overnight at 37 °C in 5 % CO_2_. Bacterial cultures treated with rifampin (1 μg/mL; Sigma-Aldrich) were used as a positive control for growth inhibition. Middlebrook 7H9 alone was used as a negative control. The samples were read using a spectrophotometer at 570 nm.

### Chromatographic and immunological assays

Total oxidant status was assessed using an enzymatic assay kit from Rel Assay Diagnostics (Gaziantep, Turkey) following the manufacturer’s protocol. The results are expressed in terms of micro molar hydrogen peroxide equivalent per liter (μmol H_2_O_2_ Equiv./L). Total antioxidant status was measured using the Antioxidant Assay kit from Cayman Chemical (Ann Harbor, MI). In this assay, the capacity of the antioxidants to prevent ABTS (2,2′-azino-di-[3-ethylbenzthiazoline sulphonate]) oxidation is compared with that of Trolox, a water-soluble tocopherol analogue, and is quantified as molar Trolox equivalents. Lipid peroxidation in plasma and culture supernatants was quantified using an assay kit from Cayman Chemical, which measures the formation of malondialdehyde (MDA). DNA/RNA oxidation was measured in culture supernatants using an immunoassay from Cayman Chemical that detects all three oxidized guanine species: 8-hydroxy-2′-deoxyguanosine from DNA, 8-hydroxyguanosine from RNA, and 8-hydroxyguanine from either DNA or RNA. Results from this assay were shown as concentration of 8-OH-DG. Cell death was estimated by quantification of lactate dehydrogenase (LDH) in culture supernatants using a kit from Cayman Chemical following the manufacturer’s protocol. Cell death was plotted as percentage of cell death compared with positive control (H_2_O_2_). Intracellular production of ROS in primary human macrophages was assessed by staining cells with the oxidative fluorescent dye probe, dihydroethidium (DHE) 5 mM (Invitrogen/Molecular Probes, Grand Island, NY) for 30 min at 37 °C in 5 % CO_2_ and then analyzed using a flow cytometer. The cells used for the ROS measurement assay were previously detached from the culture plates using trypsin 0.25 %, washed and resuspended in phenol and serum free medium. Results were plotted as histograms where the mean fluorescence intensity (MFI) was compared between the experimental groups.

### In vivo experiments

Mice were anesthetized using ketamine (Vetbrands, Brazil; 100 mg/kg) and xylazine (Vetbrands; 15 mg/kg), diluted in sterile saline and administrated intraperitoneally. Mice were infected with H37Rv *M. tuberculosis* strain (approx. 3 × 10^4^ bacilli) inoculated intratracheally as described previously [30]. Mice were given NAC (400 mg/kg) by gavage daily for 6 days. The dose of NAC used was based on studies reported previously, which describe the effect of NAC treatment during chronic Mtb infection in vivo, as well as in other experimental models [23, 36–38]. On day 7 of infection, mice were euthanized using CO_2_ exposure chamber, followed by cervical dislocation, before lungs were harvested. For CFU counting, lungs were homogenized in 1 mL of cold PBS using a cell strainer (100 μm; Corning, USA) and plated in Middelbrook 7H11 medium (Difco) agar plate.

### Statistical analysis

The median values with interquartile ranges were used as measures of central tendency. For in vitro experiments, bars represent mean and standard errors. The Mann-Whitney test (for two groups) or Kruskal-Wallis with Dunn’s multiple comparisons or linear trend post-hoc tests (for more than two groups) were used to compare continuous variables. Differences observed in the experimental studies were assessed using Student’s *t*-test (for comparisons between two groups) or One-Way ANOVA with Tuckey post-test. A p-value of <0.05 was considered statistically significant.

## Results and discussion

### NAC inhibits oxidative stress, lipid peroxidation, DNA oxidation and cell death in M. tuberculosis-infected human macrophages

Previous studies in active TB patients point to an association of this disease with excessive oxidative stress by demonstrating decreased systemic concentrations of antioxidants and enhanced spontaneous generation of free radicals compared to individuals without TB [27, 39]. The measurement of total oxidant status, total antioxidant status and lipid peroxidation offers a reliable way to verify that there is an imbalance between the production of free radicals and the ability of the body to detoxify their effects through neutralization by antioxidants. This imbalance of oxidative stress status results in irreversible cell damage, leading to many pathophysiological conditions [40–44]. To characterize the oxidative stress status during TB infection we measured total oxidant status, total antioxidant status and lipid peroxidation in the plasma of pulmonary TB (PTB) patients and those with latent *M. tuberculosis* infection (LTBI) or healthy uninfected individuals. Lipid peroxidation, DNA oxidation and cell death, as well as accumulation of reactive oxygen species (ROS) were also assessed in cultures of primary human monocyte-derived macrophages infected with *M. tuberculosis* and treated or not with NAC.

**Fig. 1:**
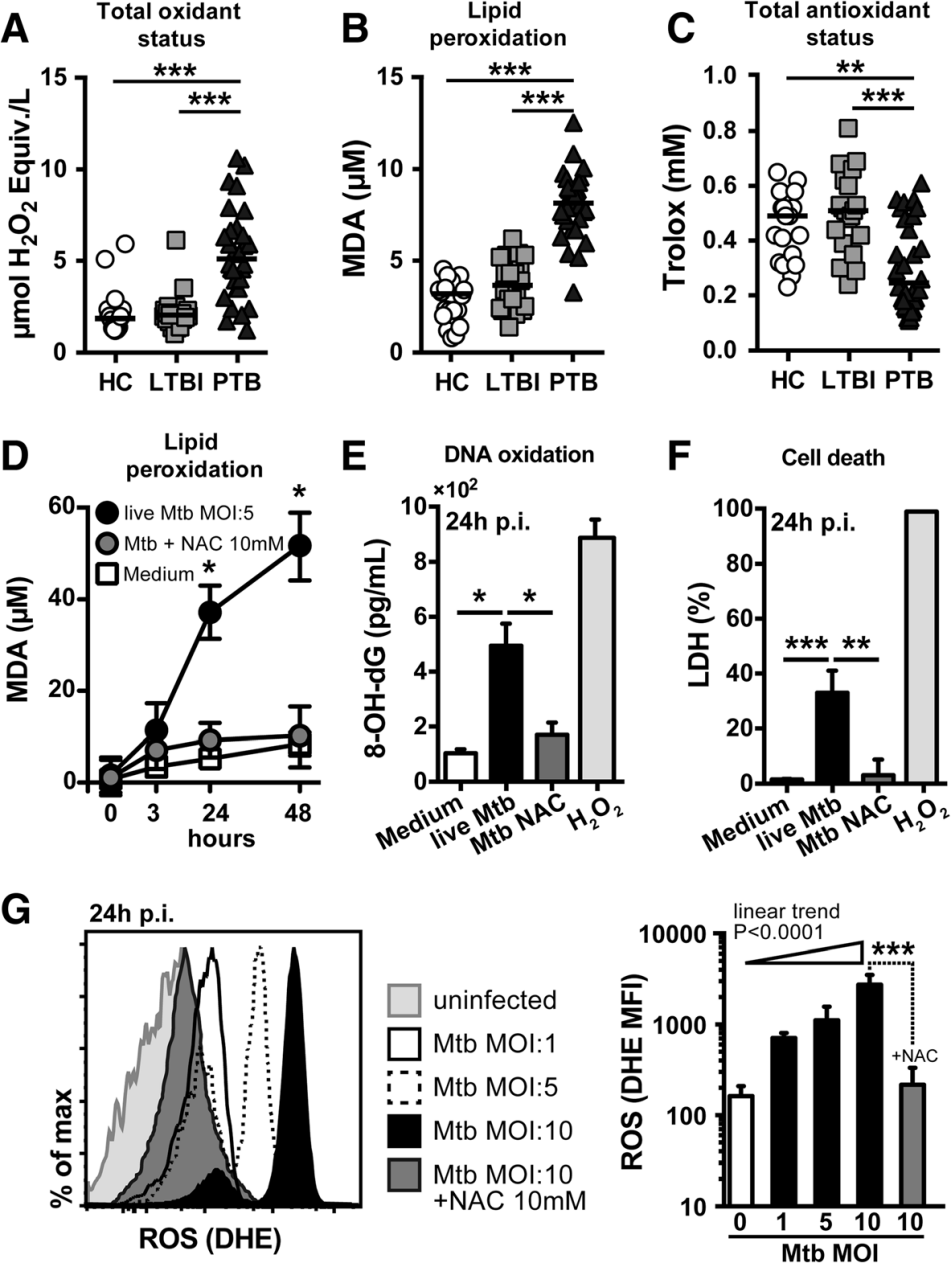
NAC reverts *M. tuberculosis*-induced oxidative stress. Cryopreserved heparinized plasma samples collected from active pulmonary TB (PTB; *n* = 30), latent TB individuals (LTBI; *n* = 20) and healthy controls (HC; *n* = 20) from Salvador Brazil were used in these studies. Total oxidant status (**a**), lipid peroxidation (**b**) and total antioxidant status (**c**) were measured as described in Methods. **d-f** Primary human monocyte-derived macrophages were infected with H37Rv *M. tuberculosis* at MOI of 5 and treated or not with NAC (10 mM). Lipid peroxidation (**d**), DNA oxidation (**e**) and cell death (**f**) were assessed at indicated time points post-infection (p.i.) as described in Methods. **g** Intracellular production of ROS in primary human macrophages infected with H37Rv *M. tuberculosis* at different MOI was measured by flow cytometry. ROS production was also verified in infected-macrophages after NAC treatment (10 mM). Results were plotted as histograms where the mean fluorescence intensity (MFI) was compared between the experimental groups. In **a-c** lines represent median values. In **d-g** data represent means ± SEM of triplicate samples from a minimum of six donors. In **a-f** data were analyzed using Kruskal-Wallis test with Dunn’s multiple comparisons post-test. In **g** MOI titration data were compared using Kruskal-Wallis test with non-parametric linear trend post-test. The effect of NAC treatment was analyzed using the Mann-Whitney *U* test. The data shown are representative of three independent experiments. (*p <0.05; **p <0.01, ***p < 0.001)

Interestingly, PTB patients exhibited substantial elevation of total oxidation status (Fig. 1a) and lipid peroxidation (Fig. 1b) in the plasma while simultaneously displaying a significant reduction in soluble antioxidants (Fig. 1c) compared to LTBI cases or uninfected controls (Fig. 1a-c). Following *M. tuberculosis* infection in vitro, human monocyte-derived macrophages displayed augmented lipid peroxidation (Fig. 1d), DNA oxidation (Fig. 1e) and cell death induction (Fig. 1f), which were accompanied by a dramatic accumulation of intracellular ROS (Fig. 1g). In our experiments we found that treatment of *M. tuberculosis*-infected macrophage cultures with NAC (10 mM) significantly decreased ROS accumulation, lipid peroxidation and DNA oxidation, while restoring cell viability (Fig. 1d-g). Together these findings support the concepts that TB is associated with excessive oxidative stress and death in infected macrophages and show that this response can be successfully reduced by treatment with NAC.

### NAC limits mycobacterial extracellular growth independent of pH

**Fig. 2:**
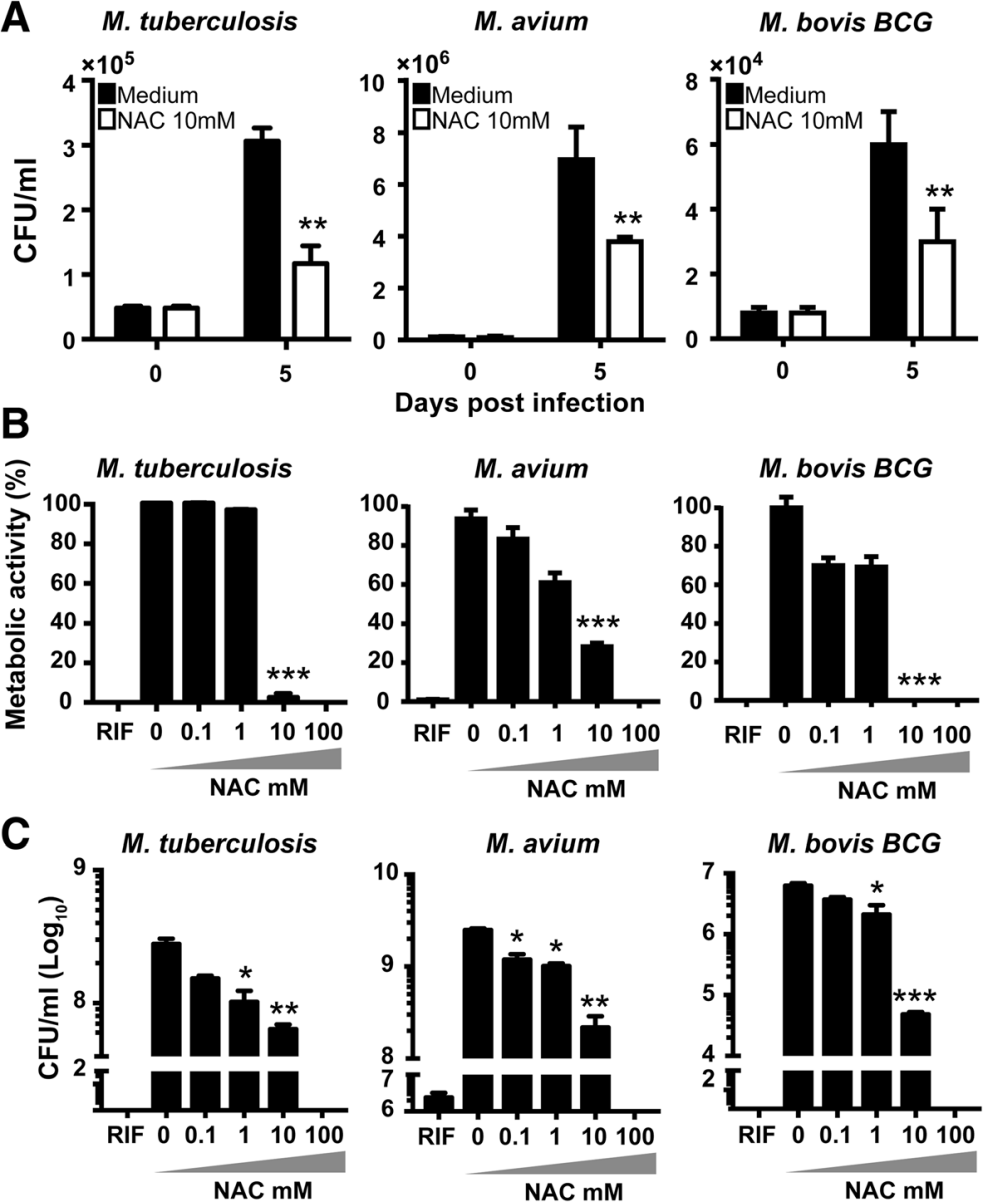
NAC restrains mycobacterial growth within THP-1 macrophages and exhibits a direct anti-mycobacterial effect on extracellular bacteria in vitro. **a** Human-THP-1 macrophages were infected with *M. tuberculosis*, *M. avium* or *M. bovis* strains at an MOI of 10 for 3 h. Extracellular bacteria were removed by washing. Cells were then cultivated for 5 days in the presence of NAC at 10 mM. CFU counts were assessed as described in Methods. **b** and **c** Mycobacteria strains were grown in Middlebrook 7H9 supplemented with OADC in 96-well plates. Metabolic activity measurements (**b**) and CFU counts (**c**) were performed as described in Methods. Significant differences were observed for the indicated experimental conditions compared to untreated cultures (*p < 0.05; **p < 0.01, ***p < 0.001). The data represent the means ± SEM of triplicate samples. The data shown are representative of three independent experiments

A growing body of evidence suggests that NAC can affect bacterial viability inside of eukaryotic host cells [45]. To test this concept with mycobacteria we infected THP-1 cells (to reduce variability between blood donors) with *M. tuberculosis*, *M. avium* or *M. bovis BCG* bacilli (MOI of 10) for 3 h, replaced the media, and added NAC (10 mM) to half of the cultures. Intracellular bacterial growth was then assessed 5 days later by lysing the cells and measuring CFU. We observed a marked reduction in bacterial loads for each of the mycobacterial species in the NAC-treated cultures demonstrating the ability of the drug to limit mycobacterial growth within THP-1 cells (Fig. 2a). Although minor bacterial growth occurred in presence of NAC it was clearly inhibited when it was compared to control (untreated cells).

**Fig. 3:**
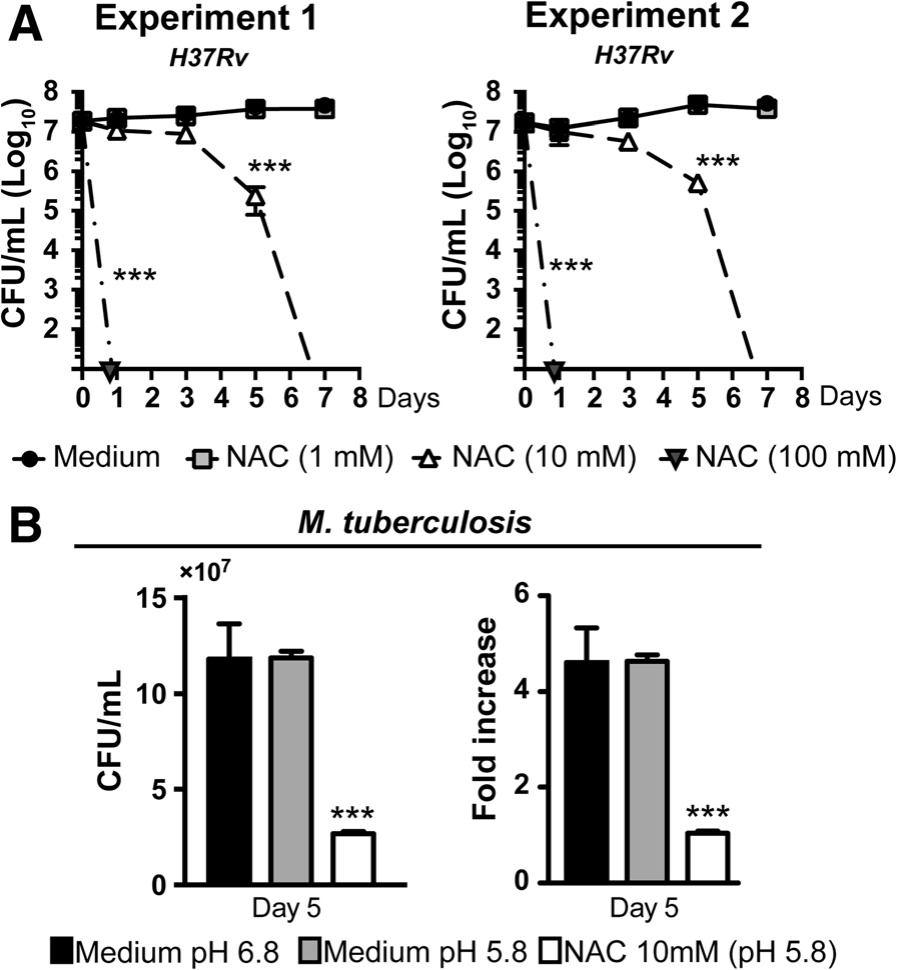
NAC limits mycobacterial proliferation by acting as an anti-mycobacterial compound. *Mycobacterium tuberculosis* was grown in Middlebrook 7H9 supplemented with OADC as described in Methods. **a** Kinetic of mycobacteria growth during 7 days in the presence of NAC at indicated concentrations was verified using CFU counts. **b** Mycobacterial growth was evaluated at different pH as described in Methods. Fold increase of bacterial growth was calculated as the ratio of CFU number counted on days 0 and 5. Significant differences were observed for the indicated experimental conditions (***p < 0.001). The data represent the means ± SEM of triplicate samples. The data shown are representative of at least three independent experiments

High concentrations of NAC have been shown to directly inhibit extracellular growth of a number of bacterial species [24, 25, 46]. Indeed, NAC inhibits biofilm formation of a variety of medically important gram-positive and gram-negative bacteria, e.g. *P. aeruginosa, K. pneumoniae, S. epidermidis, S. aureus* and *Escherichia coli* [25, 26, 46–48]. Interestingly, NAC does not inhibit growth of methicillin-sensitive and resistant *S. aureus* strains at 49 mM (8 mg/mL) [49], suggesting that bacterial species may diverge in susceptibility to the effects of this drug. Here, we tested whether NAC could affect the growth of different strains of mycobacteria in cell free culture broth. Bacterial metabolic activity and CFU numbers were reduced in a dose dependent manner at day 5 following treatment with NAC (Fig. 2b and c). Moreover, a hypervirulent *M. tuberculosis* Beijing strain (Beijing 1471) [30, 50] was also highly susceptible to NAC treatment (Additional file 1: Figure S1). Next, we performed a kinetic experiment in which *M. tuberculosis* was cultured in the presence or absence of NAC in a wide range of concentrations for different days and bacterial growth was assessed by CFU counts. We observed a striking >2log_10_ reduction in CFU counts in cultures treated with NAC 10 mM (Fig. 3a) at 5 days and mycobacterial sterilization in the cultures after 7 days of treatment. These findings demonstrate that NAC exhibits a potent anti-mycobacterial effect while dampening infection-induced oxidative stress, promoting increased survival of infected cells.

In our experimental conditions, we observed that the pH of cellular or mycobacterial growth media dropped in the presence of NAC (from 6.8 to 5.8) at 10 mM. We tested whether the doses of NAC used to demonstrate inhibition of mycobacterial growth in vitro maybe toxic for human cells. Uninfected human macrophage-like THP-1 cells were cultured for 24 h in the presence of different concentrations of NAC in media with or without pH adjustment (pH = 7.4 in adjusted conditions and pH = 5.8 in unadjusted conditions). Cell viability was assessed by flow cytometry (using frequency of cells staining positive for UV live/dead). We observed that NAC did not affect THP-1 cell viability at 10 mM, but exhibited significant pH dependent cytotoxicity at higher concentrations such as 100 mM (Additional file 2: Figure S2A).

A low pH of medium can potentially limit the growth of some bacteria in vitro [51]. However, mycobacterial species have been reported to grow under acidic conditions in complex media [52]. To test whether the modification of cell culture pH by addition of NAC could explain the changes in mycobacterial growth observed here, we cultivated mycobacterial strains in media with either acidic pH (pH = 5.8) or neutral pH (pH ~7.4). Importantly, the pH adjustment of the treated mycobacterial cultures only partially reduced the capacity of NAC to inhibit the metabolic activity of the bacilli and the bacteria growth (Additional file 2: Figure S2B and C). Of note, acidification of bacterial medium, to a pH similar to what is observed during NAC treatment, did not affect *M. tuberculosis* growth (Fig. 3b). These results argue that NAC has direct antimicrobial activity that compromises mycobacterial growth independent of acidification of culture pH.

### NAC promotes reduction of bacterial burden in infected mice

**Fig. 4:**
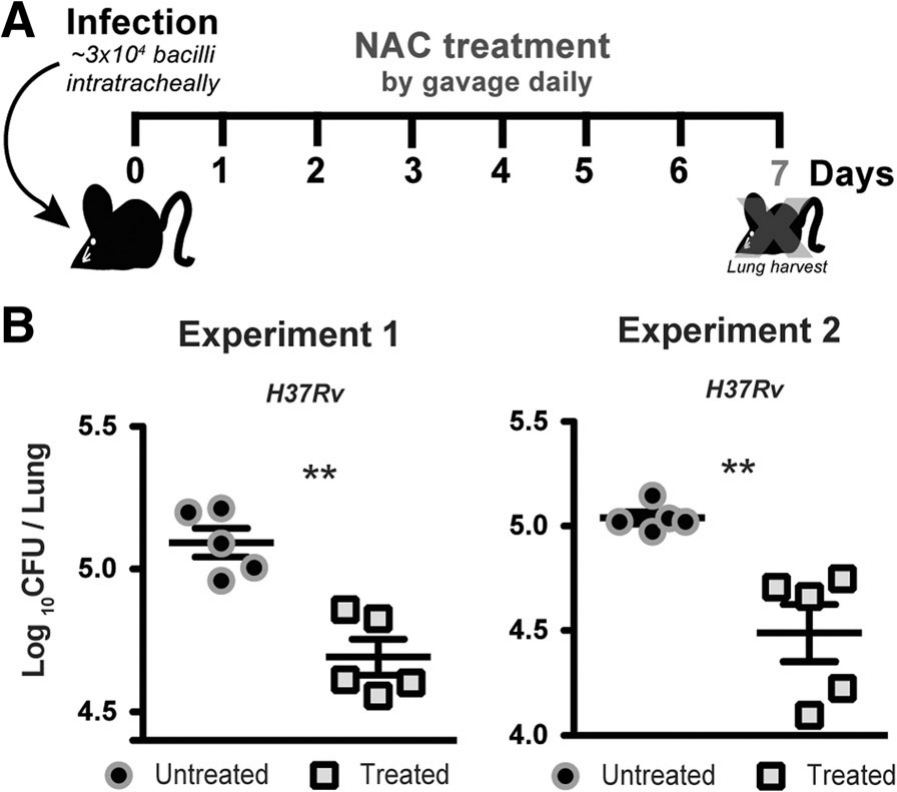
Short-term NAC treatment results in a significant reduction of mycobacterial loads in the lungs from mice infected with *M. tuberculosis*. **a** C57BL/6 mice were intratracheally infected with ~ 3 × 10^4^ bacilli of H37Rv strain. Mice were treated or not with NAC (400 mg/kg) by gavage daily for 6 days. **b** On day 7 p.i., animals were euthanized and lungs were harvested. CFU/g values in lungs were determined as described in Methods. Data represent individual values and means ± SEM from a total of 5 animals per group. Data from two independent experiments are shown. Significant differences were observed for the indicated groups (**p < 0.01)

We further examined whether NAC exhibits anti-mycobacterial activity in vivo. C57BL/6 mice were intratracheally infected with *M. tuberculosis* (~3 × 10^4^ forms) and then treated with NAC (400 mg/kg daily) via gavage starting on day 1 of infection, and continuing for 6 days (Fig. 4a). Bacterial burden was measured on day 7 post-infection to evaluate any potential early bactericidal activity (EBA) in vivo. Interestingly, short-term NAC treatment of mice infected with H37Rv *M. tuberculosis* in vivo resulted in a significant reduction of mycobacterial loads in the lungs compared to those of untreated animals (Fig. 4b), suggesting that NAC is able to limit mycobacterial growth in vivo. In agreement with these findings, a recent study revealed that oral treatment of *M. tuberculosis*-infected guinea pigs with NAC for up to 60 days post-infection reduces both bacterial burden and extra-pulmonary disease severity [23]. Nevertheless, the effects of NAC in that study were attributed solely to its antioxidant capacity whereas we observed an additional direct anti-mycobacterial effect of NAC treatment. Whether the same dual effects of NAC treatment would be observed in NAC treated TB patient remains to be investigated.

### The antimicrobial activity of NAC against mycobacteria is not dependent on the host NADPH oxidase system

**Fig. 5:**
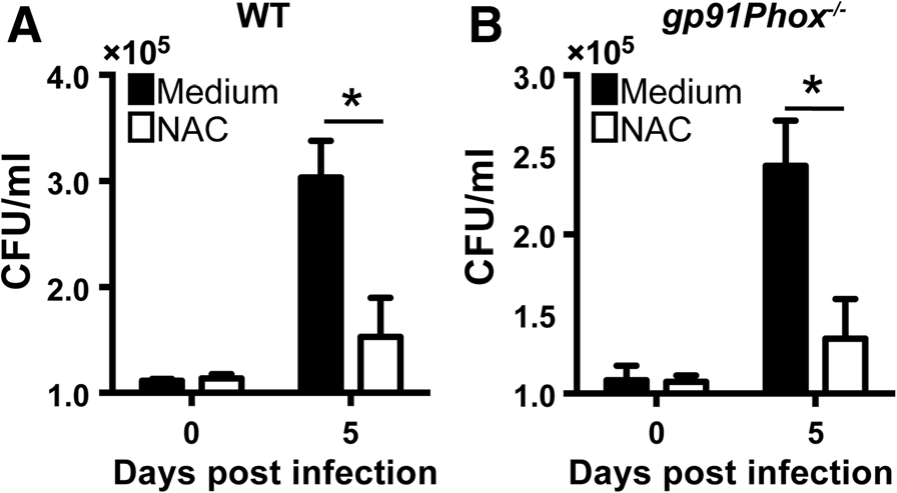
Anti-mycobacterial property of NAC occurs independently of the host NADPH oxidase system. BMDMs generated from **a** C57BL/6 and **b** *gp91Phox* ^*−/−*^ mice were infected with *M. tuberculosis* and intracellular growth of the bacteria was assessed as described in Methods. The data represent the means ± SEM of triplicate samples. Significant differences were observed for the indicated groups (*p < 0.05). The data shown are representative of at least two independent experiments

NAC is a known antioxidant and is commonly used as a ROS scavenger in diverse scenarios [2]. NADPH oxidase is a major source of intracellular ROS in eukaryotic cells [28, 29]. To determine whether NAC acts independently of NADPH-derived ROS inside macrophages we infected gp91Phox^−/−^ deficient mouse macrophages, which lack the main NADPH subunit and thus lack NADPH activity [29], with *M. tuberculosis* in the presence or absence of non-host cell cytotoxic doses of the compound (10 mM). We observed that treatment with NAC inhibited bacteria proliferation in both wild type and gp91Phox^−/−^ macrophages (Fig. 5a and b, respectively), indicating that the antimicrobial activity of NAC is not dependent on a fully functional host NADPH oxidase system.

NAC is a precursor of both the amino acid L-cysteine and of reduced glutathione (GSH) [2]. In a previous study of HIV-infected patients, in vitro NAC treatment led to increased levels of GSH inside macrophages and subsequent infection of these macrophages with *M. tuberculosis* resulted in reduction of intracellular bacterial counts [53]. Once produced, GSH has been described to limit bacterial growth directly through a mechanism dependent on the mycobacterial enzyme gamma-glutamyl transpeptidase [54]. This enzyme cleaves GSH and S-nitrosoglutathione to form the dipeptide cysteinylglycine (Cys-Gly), which is transported to the interior of the bacterial cell by the multicomponent ABC transporter dipeptide permease [54, 55]. In addition, a mutant *M. tuberculosis* strain lacking expression of gamma-glutamyl transpeptidase has been shown to grow normally in the presence of GSH [54]. Therefore, one possibility on how NAC is involved in anti-mycobacterial effects of pathogenic mycobacteria is that NAC may exert a bactericidal effect on mycobacteria through the generation of GSH and in turn Cys-Gly. Additional studies using mycobacteria deficient in gamma-glutamyl transpeptidase could be used to test this hypothesis.

## Conclusions

The observation that NAC exerts anti-mycobacterial activity in vivo has previously been interpreted as resulting solely from the anti-oxidant properties of the compound. In light of our discovery of a direct antimicrobial function of NAC against different mycobacterial strains, this conclusion should be reassessed. Our finding that NAC possesses dual modes of action suggests that NAC warrants re-examination as a therapeutic treatment of mycobacterial infection. Shorter anti-TB treatment is needed in view of the high rate of drug toxicity, cost and complexity of the current 6-month daily regimen [56]. Accordingly, clinical trials of this potential adjunct anti-TB therapy would provide a good prospect for reducing the current long-term treatment against TB.

## Additional files

Additional file 1: Figure S1. NAC inhibits the growth of hypervirulent Beijing strain in vitro*.* Beijing 1471 *M. tuberculosis* strain was grown in Middlebrook 7H9 supplemented with OADC with or without NAC (10 mM). CFU counts were performed as described in Methods. Significant differences were observed for the indicated experimental conditions compared to untreated cultures. The data represent the means ± SEM from two independent experiments. (***p* < 0.01). (TIF 48 kb)
Additional file 2: Figure S2. NAC inhibits mycobacterial growth in vitro independent of pH. (A) Uninfected THP-1 cells were incubated with different concentrations of NAC at varying pH (acidic pH (pH 5.8) or neutral pH (pH ~7.4)). Cellular viability was assessed using live/dead staining and analyzed by flow cytometry (B and C). Mycobacteria strains grown in Middlebrook 7H9 supplemented with OADC were exposed to different concentrations of NAC at the indicated pH. Metabolic activity measurement (B) and CFU counts (C) were performed as described in Methods. Significant differences were observed for the indicated experimental conditions compared to untreated cultures (***p* < 0.01, ****p* < 0.001). The data represent the means ± SEM of triplicate samples. The data shown are representative of at least two independent experiments. (TIF 1382 kb)

## Abbreviations

ABTS: 2,2′-azino-di-[3-ethylbenzthiazoline sulphonate]
AFB: Acid-fast bacilli
BMDM: Bone marrow derived macrophages
CFU: Colony-forming units
COPD: Chronic obstructive pulmonary disease
Cys-Gly: Cysteinylglycine
DHE: Dihydroethidium
EBA: Early bactericidal activity
GSH: Glutathione
IQR: Interquartile range
LDH: Lactate dehydrogenase
LTBI: Latent *M. tuberculosis* infection
MDA: Malondialdehyde
MFI: Mean fluorescence intensity
NAC: N-acetyl-cysteine
NF-κB: Nuclear factor-κB
PTB: Pulmonary TB
ROS: Reactive oxygen species
TB: Tuberculosis
TST: Tuberculin skin test
