# Supplementary figures and images for: N-acetyl-cysteine exhibits potent anti-mycobacterial activity in addition to its known anti-oxidative functions
Source: BMC Microbiol. 2016 Oct 28;16:251. doi: 10.1186/s12866-016-0872-7 (PMC5084440; doi:10.1186/s12866-016-0872-7)

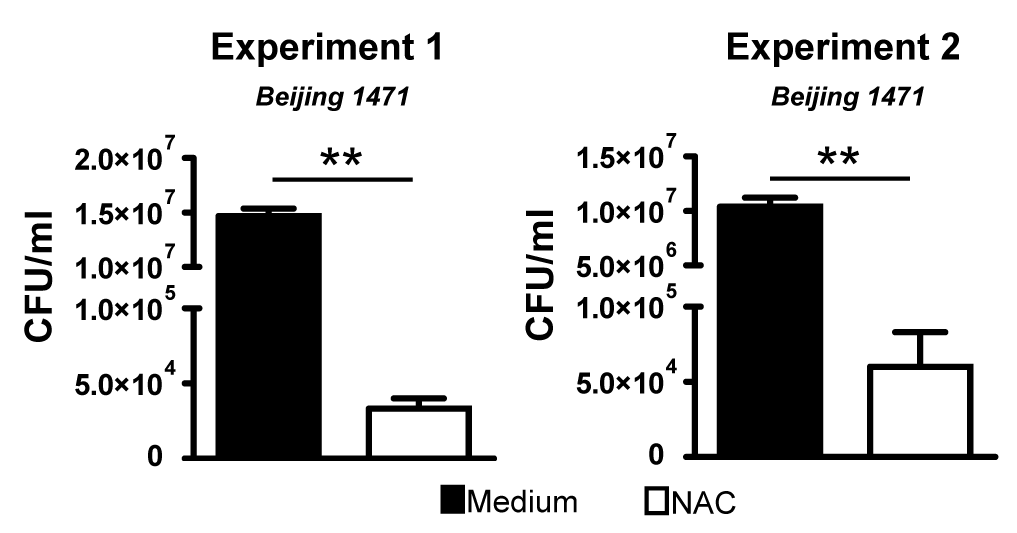

Supplement: Additional file 1: Figure S1. — NAC inhibits the growth of hypervirulent Beijing strain in vitro. Beijing 1471 M. tuberculosis strain was grown in Middlebrook 7H9 supplemented with OADC with or without NAC (10 mM). CFU counts were performed as described in Methods. Significant differences were observed for the indicated experimental conditions compared to untreated cultures. The data represent the means ± SEM from two independent experiments. (**p < 0.01). (TIF 48 kb) [file 12866_2016_872_MOESM1_ESM.tif]

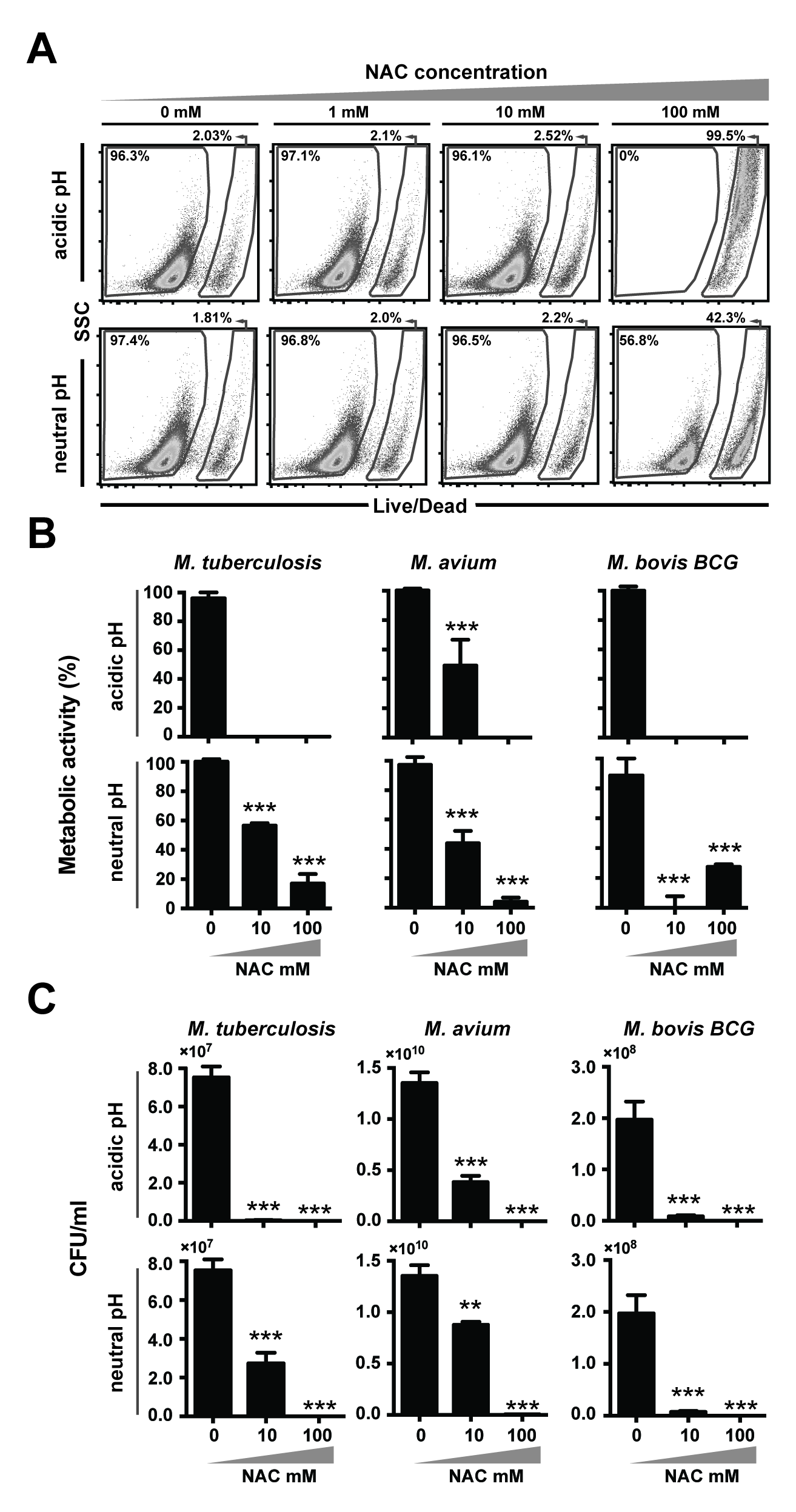

Supplement: Additional file 2: Figure S2. — NAC inhibits mycobacterial growth in vitro independent of pH. (A) Uninfected THP-1 cells were incubated with different concentrations of NAC at varying pH (acidic pH (pH 5.8) or neutral pH (pH ~7.4)). Cellular viability was assessed using live/dead staining and analyzed by flow cytometry (B and C). Mycobacteria strains grown in Middlebrook 7H9 supplemented with OADC were exposed to different concentrations of NAC at the indicated pH. Metabolic activity measurement (B) and CFU counts (C) were performed as described in Methods. Significant differences were observed for the indicated experimental conditions compared to untreated cultures (**p < 0.01, ***p < 0.001). The data represent the means ± SEM of triplicate samples. The data shown are representative of at least two independent experiments. (TIF 1382 kb) [file 12866_2016_872_MOESM2_ESM.tif]
